# Antibiotic use in poultry: a survey of eight farms in Thailand

**DOI:** 10.2471/BLT.17.195834

**Published:** 2017-11-27

**Authors:** Gumphol Wongsuvan, Vanaporn Wuthiekanun, Soawapak Hinjoy, Nicholas PJ Day, Direk Limmathurotsakul

**Affiliations:** a Mahidol–Oxford Tropical Medicine Research Unit, Faculty of Tropical Medicine, Mahidol University, 420/6 Rajvithi Road, Bangkok, 10400, Thailand.; b Department of Disease Control, Ministry of Public Health, Nonthaburi, Thailand.; c Centre for Tropical Medicine and Global Health, University of Oxford, Oxford, England.

## Abstract

**Objective:**

To investigate antibiotic use in poultry farms in Thailand and estimate the total amount of antibiotics used annually in Thai production of chicken meat.

**Methods:**

In a single province, we surveyed eight farms in which chickens were raised for meat and interviewed the farms’ owners in 2016. The antibiotic use for each chicken was defined as the amount of antibiotic given to the chicken over its entire lifetime divided by the target weight of the chicken at the time of its slaughter. Assuming that the results were nationally representative, we estimated annual antibiotic use on all Thai chickens raised for meat.

**Findings:**

No use of antibiotics for growth promotion was reported. Five farms raised 1-kg chickens for company A and reportedly used no antibiotics unless the chickens were sick. The other three farms raised 3-kg chickens for company B and reported routine use of antibiotics for prophylaxis. Per kg final weight, each chicken raised for company B was reportedly routinely given a mean of 101 mg of antibiotics – that is, 33 mg of amoxicillin, 29 mg colistin, 19 mg oxytetracycline, 18 mg doxycycline and 2 mg tilmicosin. The total amount of antibiotic used on all Thai chickens raised for meat in 2016 was estimated to be 161 tonnes.

**Conclusion:**

Each year in Thailand, many tonnes of antibiotics are probably routinely used in raising chickens for meat. Labels on retail packs of meat should include data on antibiotic use in the production of the meat.

## Résumé

**Objectif:**

Analyser l'utilisation d'antibiotiques dans les élevages de poulets destinés à la production de viande en Thaïlande et estimer la quantité totale d'antibiotiques utilisés annuellement à cette fin dans le pays.

**Méthodes:**

Dans une province, en 2016, nous avons enquêté dans huit élevages de poulets destinés à la production de viande et avons interrogé les propriétaires. L'utilisation d'antibiotiques, pour chaque poulet, était définie comme la quantité d'antibiotiques administrée à l'animal pendant toute sa durée de vie divisée par son poids cible au moment de l'abattage. Partant du principe que les résultats étaient représentatifs de la situation nationale, nous avons estimé la quantité annuelle d'antibiotiques utilisée pour l'ensemble des poulets thaïlandais destinés à la production de viande.

**Résultats:**

Aucune utilisation d'antibiotiques visant à stimuler la croissance n'a été rapportée. Cinq élevages produisaient des poulets de 1 kg pour la société A et ont indiqué ne recourir aux antibiotiques que lorsque les poulets étaient malades. Les trois autres élevages produisaient des poulets de 3 kg pour la société B et ont indiqué utiliser régulièrement des antibiotiques en prévention. Par kg de poids final, chaque poulet élevé pour la société B recevait régulièrement une moyenne de 101 mg d'antibiotiques: 33 mg d'amoxicilline, 29 mg de colistine, 19 mg d'oxytétracycline, 18 mg de doxycycline et 2 mg de tilmicosine. La quantité totale d'antibiotiques utilisée pour l'ensemble des poulets thaïlandais destinés à la production de viande en 2016 était estimée à 161 tonnes.

**Conclusion:**

Chaque année, en Thaïlande, plusieurs tonnes d'antibiotiques sont probablement utilisées de façon régulière dans les élevages de poulets destinés à la consommation. Les étiquettes apposées sur les emballages de vente au détail de la viande devraient inclure des informations sur les antibiotiques utilisés dans la production.

## Resumen

**Objetivo:**

Investigar el uso de antibióticos en la producción de carne de pollo en Tailandia y estimar la cantidad total de antibióticos que se utilizan anualmente en la producción tailandesa de dicha carne.

**Métodos:**

En una sola provincia, realizamos una encuesta en ocho granjas en las que se criaban pollos para obtener carne y entrevistamos a los propietarios de las granjas en 2016. El uso de antibióticos para cada pollo se definió como la cantidad de antibióticos administrados al animal a lo largo de su vida, dividido por su peso objetivo en el momento de sacrificarlo. Suponiendo que los resultados fueran representativos a nivel nacional, estimamos el uso anual de antibióticos en todos los pollos tailandeses criados para obtener carne.

**Resultados:**

No se registró el uso de antibióticos para promover el crecimiento. Cinco granjas criaban pollos de 1 kg para la empresa A y, según los informes, no usaban antibióticos a menos que los pollos estuvieran enfermos. Las otras tres granjas criaban pollos de 3 kg para la empresa B e informaron del uso rutinario de antibióticos para la profilaxis. Por cada kilo de peso final, cada pollo criado para la empresa B recibía de forma rutinaria una media de 101 mg de antibióticos, es decir, 33 mg de amoxicilina, 29 mg de colistina, 19 mg de oxitetraciclina, 18 mg de doxiciclina y 2 mg de tilmicosina. La cantidad total de antibióticos utilizados en todos los pollos tailandeses criados para obtener carne en 2016 se estimó en 161 toneladas.

**Conclusión:**

Cada año, en Tailandia, se usan muchas toneladas de antibióticos de forma rutinaria en la cría de pollos para obtener carne. Las etiquetas de los paquetes de carne al por menor deberían incluir datos sobre el uso de antibióticos en la producción de la carne.

## ملخص

**الغرض:**

التحقيق في موضوع استخدام المضادات الحيوية في إنتاج لحوم الدواجن في تايلند وتقدير الكمية الإجمالية للمضادات الحيوية المستخدمة سنويًا في الإنتاج التايلندي لمثل هذا النوع من اللحوم.

**الطريقة:**

قمنا في إحدى المقاطعات بإجراء مسح يشمل ثماني مزارع حيث كانت تتم تربية الدواجن بغرض الاستفادة من لحومها، وقمنا بإجراء مقابلات مع مالكي المزارع في عام 2016. وقد تم تعريف استخدام المضادات الحيوية لكل دجاجة باعتباره كمية المضادات الحيوية المقدمة للدجاجة على مدار عمرها بالكامل مقسومة على الوزن المستهدف للدجاجة عند ذبحها. وبافتراض أن النتائج كانت نتائج تمثيلية على الصعيد الوطني، فقد قمنا بتقدير كمية المضادات الحيوية المستخدمة سنويًا لجميع الدواجن التي تمت تربيتها بغرض الاستفادة من لحومها في تايلند.

**النتائج:**

لم يتم الإبلاغ عن استخدام المضادات الحيوية لتسريع نمو الدواجن. قامت خمس مزارع بتربية الدواجن بوزن 1 كجم من أجل الشركة (أ) وأفادت التقارير أنها لم تستخدم أية مضادات حيوية إلا في حالة مرض الدواجن. وقد قامت المزارع الثلاث الأخرى بتربية الدواجن بوزن 3 كجم من أجل الشركة (ب) وأبلغت عن قيامها بالاستخدام الروتيني للمضادات الحيوية لتكون بمثابة العلاج الوقائي للدواجن. لقد أفادت التقارير أن كل دجاجة تمت تربيتها من أجل الشركة (ب) تم إعطاؤها مقدارًا يبلغ 101 ملجم من المضادات الحيوية بصورة روتينية لكل كجم من وزن الدجاجة النهائي – أي 33 ملجم من الأموكسيسيلين، و29 ملجم من الكوليستين، و19 ملجم من الأوكسيتيتراسيكلين، و18 ملجم من الدوكسيسايكلين، و2 ملجم من التلميكوسين. تم تقدير الكمية الإجمالية من المضادات الحيوية المستخدمة على جميع الدواجن التي تمت تربيتها للاستفادة من لحومها بتايلند في عام 2016 بمقدار 161 طنًا.

**الاستنتاج:**

على المرجح أنه يتم في تايلند سنويًا استخدام العديد من الأطنان من المضادات الحيوية بصورة روتينية في تربية الدواجن للاستفادة من لحومها. يجب على الملصقات الموجودة على عبوات البيع بالتجزئة للحوم الدواجن أن تحتوي على البيانات الخاصة باستخدام المضادات الحيوية في إنتاج هذه اللحوم. ****

## 摘要

**目的:**

旨在评估性与性别认同少数群体中由于性取向和性别认同感知而引发的人身暴力和性暴力的盛行率。

**方法:**

我们针对 2000 年至 2016 年 4 月发表的同行评审和灰色文献，搜索了 9 个没有语言限制的数据库。我们纳入了超过 50 名参与者的研究，这些参与者被认为是由于性取向、性别认同或性别表达引发的人身暴力和性暴力。我们排除了亲密伴侣的暴力和自我伤害。由于大多数研究的异质性和置信区间的缺失，我们没有进行元分析。

**结果:**

我们收录了 50 个国家的 76 篇文章。这些研究涵盖了 1995 至 2014 年间进行的 74 项研究，其中包括总计 202 607 名性与性别认同少数参与者。由于缺乏标准化的措施，有时是小样本和非随机样本，因此数据质量相对较差。在研究中，所有性与性别认同少数群体作为一类整体来研究，人身暴力和性暴力的盛行率分别从 6%（一项包括 240 人的研究中）到 25%（196 人中 49 人）和 5.6% (28/504) 到 11.4% (55/484)。对于变性人来说，盛行率分别从 11.8%（34 人的子样本）到 68.2% (75/110) 和 7.0%（包含 255 人的研究）到 49.1% (54/110)。

**结论:**

需要更多有关不同地理和文化背景下由于性取向和性别认同感知而引发的人身暴力和性暴力的盛行率、风险因素和后果的数据。国家预防暴力的政策和干预措施应该将性与性别认同少数群体包括在内。

## Резюме

**Цель:**

Изучить использование антибиотиков при выращивании мясных пород кур в Таиланде и оценить общее количество антибиотиков, используемых ежегодно в производстве такого мяса в стране.

**Методы:**

В 2016 году в одной провинции были обследованы восемь ферм, в которых цыплята выращивались на мясо, и опрошены владельцы фермерских хозяйств. Использование антибиотиков в расчете на каждого цыпленка определяли как количество антибиотика, получаемого цыпленком на протяжении всей жизни, разделенное на целевой вес цыпленка во время его убоя. Предполагая, что полученные результаты были репрезентативными на национальном уровне, авторы оценили ежегодное использование антибиотиков в расчете на всех тайских цыплят, выращиваемых на мясо.

**Результаты:**

Не сообщается о применении антибиотиков для стимулирования роста. Пять ферм выращивали цыплят весом 1 кг для компании А и, по имеющимся сообщениям, не использовали никаких антибиотиков, если цыплята были здоровы. Три другие фермы выращивали цыплят весом 3 кг для компании B и сообщали о регулярном использовании антибиотиков в профилактических целях. В расчете на килограмм конечного веса каждый цыпленок, выращенный для компании B, по имеющимся сообщениям, регулярно получал в среднем 101 мг антибиотиков: 33 мг амоксициллина, 29 мг колистина, 19 мг окситетрациклина, 18 мг доксициклина и 2 мг тилмикозина. Общее количество антибиотиков, которое было использовано при выращивании мясных пород кур в 2016 году, оценивалось в 161 тонну.

**Вывод:**

По-видимому, каждый год в Таиланде при выращивании цыплят на мясо регулярно используются тонны антибиотиков. Маркировка на розничных упаковках мяса должна содержать данные об использовании антибиотиков при производстве мяса.

## Introduction

The so-called antibiotic footprint has been proposed as a global tool to communicate the total magnitude of antibiotic use in humans and livestock affecting the ecological system.^1^ In low- and middle-income countries, antibiotic use is increasing as household incomes rise and antimicrobial drugs become more affordable. In the same countries, however, use of antibiotics in the health sector and over-the-counter sales of such drugs often remain poorly controlled. Reduction in the misuse and overuse of antibiotics is critical to any global attempts to reduce environmental contamination with antibiotics and the consequent emergence and spread of antibiotic-resistant bacteria.^2^

In livestock production, farmers use antibiotics for growth promotion, prophylaxis or therapy – although their use in growth promotion is being heavily discouraged worldwide.^3^ In meat production within 30 European countries in 2015, antibiotic use per so-called population correction unit, i.e. per kg of biomass produced, varied from 2.9 mg in Norway to 434.2 mg in Cyprus.^4^ In 2015, the United States Food and Drug Administration approved 41 antibiotics for use in livestock and 31 (76%) of those were deemed to be medically important.^5^ Estimations suggest that livestock fed antibiotics excrete 75–90% of those antibiotics un-metabolized and these drugs may then enter sewage systems and water sources.^6^ Waste from livestock may contain antibiotic-resistant bacteria and active antibiotics that may contaminate the environment and then foster the emergence of antibiotic resistance, in bacteria other than those to be found in living livestock and the meat produced from it.

Antibiotic use in livestock production is generally suspected to be much higher in low- and middle-income countries than in high-income countries; however, little published evidence exists to support this suspicion. For example, there are few if any published quantitative data on antibiotic use in Thai livestock production,^7^ even though Thailand is a major exporter of chicken meat. Complex economic, political and social barriers probably exist to the surveillance of antibiotic use in livestock raised in low- and middle-income countries.^8^

Here, we determined the antibiotic use for farming Thai chicken meat and estimate the total amount of antibiotics used annually in the production of such meat. We anonymized the data in terms of the province and chicken farms surveyed, the farm owners who were interviewed and the companies that the owners were supplying with chicken meat.

## Methods

### Study area

In 2016, Thailand consisted of 77 provinces, covered 513 000 km^2^ and had an estimated population of 66 million.^9^ The country is an upper-middle-income country and a major producer and exporter of chickens. In 2016, for example, Thailand produced about 1.4 billion meat chickens and about 35% of these were exported.^10^ In 2016, the value of the poultry exports, about 2.5 billion United States dollars, represented 7.4% of the total value of all of the agricultural products exported from Thailand.^10^ Production of 80% of the chickens raised for meat was controlled by 12 large companies in 2002.^11^

### Study design

In a survey in 2016, we visited rural chicken farms and interviewed the farms’ owners in a single province in Thailand. We asked the owner of each surveyed farm about the farm, the chickens raised, the animal feed used and antibiotic usage on the farm. To encourage accurate reporting, we told the interviewees that the data collected would be anonymized and that any resultant reports would not allow the study farms, province and subjects or the companies supplied by the farms to be identified.

The main aim of the study was to estimate the antibiotic use for farming chicken meat on the surveyed farms. The antibiotic use for each chicken was defined as the amount of antibiotic given to the chicken over its entire lifetime divided by the target weight of the chicken at the time of its slaughter.

We used descriptive statistics and no statistical tests were performed. To illustrate a possible use of data on antibiotic footprints on meat packaging, we developed an example based on our findings and the similar concept of providing carbon-footprint information on meat products.^12^^,^^13^ The antibiotics reportedly used on the surveyed farms were categorized according to the fifth revision of *Critically important antimicrobials for human medicine* – a report published by the World Health Organization (WHO) in 2017.^14^ This report ranked medically important antimicrobial drugs in terms of the risk, of antimicrobial resistance, posed by their use beyond human medicine.^14^

We assumed that our results were nationally representative. That is, we assumed that: (i) the proportion of surveyed farms reportedly using antibiotics routinely, for prophylaxis, was the same as the proportion of all Thai farms raising chickens for meat that used antibiotics in this way; and (ii) the prophylactic antibiotic regimen reported by our interviewees was that same as that used across Thailand. We then estimated the amount of antibiotic used annually in all the Thai farms in which chickens are raised for meat. We ignored any non-prophylactic use of antibiotics because the data we recorded on such use were relatively poor.

### Ethics

The study protocol was approved by the Ethics Committee of Mahidol University’s Faculty of Tropical Medicine and by the University of Oxford’s Oxford Tropical Research Ethics Committee. Written informed consent was obtained from each of the interviewed farm owners.

## Results

Although the owners of ten farms were invited to participate, the only owners who agreed to be interviewed were those who had had previous contact with the interviewer and, presumably in consequence, appeared to trust the interviewer. Before giving their consent, each of the eight farm owners who were interviewed wanted to be certain that any reports resulting from the interviews would hide the identity of owner, their farm and the company that the farm supplied. The owners feared that, if any of the information released led to any negative impacts on the reputations of the companies they supplied or on the wider meat market, their contracts with the companies and their relationships with their farming friends and communities could be jeopardized.

Of the farms run by the interviewed owners, five (62%) raised 1-kg meat chickens for one company – recorded as company A – and reportedly did not use any antibiotics unless the chickens were sick. The other three surveyed farms (38%) raised 3-kg meat chickens for a different company – recorded as company B – and reportedly routinely used an antibiotic regimen for disease prevention (Table 1). None of the surveyed farms reportedly used long-term low-dose antibiotics for growth promotion. No antibiotic cartons or packages were found in the five farms producing for company A. The median capacity of the eight surveyed farms was 15 000 chickens (range: 10 000–28 000). In each of several cycles per year, the farms received broiler chicks from company A or company B and spent 21 and 41 days to raise 1-kg and 3-kg chickens, respectively.

**Table 1:** Antibiotic regimen routinely used for disease prevention among 14 000 chickens raised for 41 days, from broiler chicks, on a farm in rural Thailand, 2016

Days post-receipt, dosing for entire flock**^a^**	Amounts of active ingredients used over period**^a^**
**1–4**	
85 mL tilmicosin solution each morning and 40 g doxycycline powder each evening	85 g tilmicosin and 80 g doxycycline
**9–12**	
140 g amoxicillin powder each morning and 300 g colistin 20% powder each evening	280 g amoxiciilin and 240 g colistin
**15–18**	
340 g doxycycline powder each morning	680 g doxycycline
**21–24**	
560 g amoxicillin powder each morning and 1200 g colistin 20% powder each evening	1120 g amoxicillin and 960 g colistin
**28–31**	
4000 g oxytetracycline powder once daily	800 g oxytetracycline
**1–41**	4245 g of all antibiotics

^a^ The antibiotics were purchased either as powders, containing 500 mg amoxicillin per gram powder, 200 mg colistin per gram powder, 500 mg doxycycline per gram powder or 50 mg oxytetracycline per gram powder, or as a solution containing 250 mg tilmicosin/mL.

**Fig. 1:**
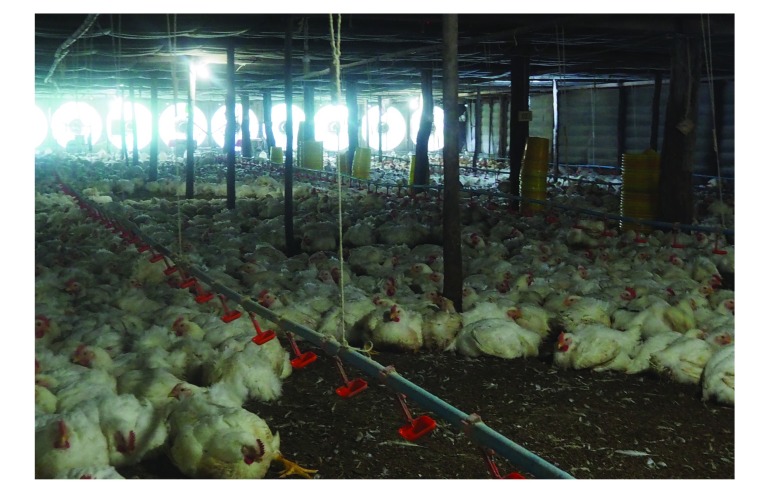
Housing for 14 000 chickens in rural farm, Thailand, 2016

**Fig. 2:**
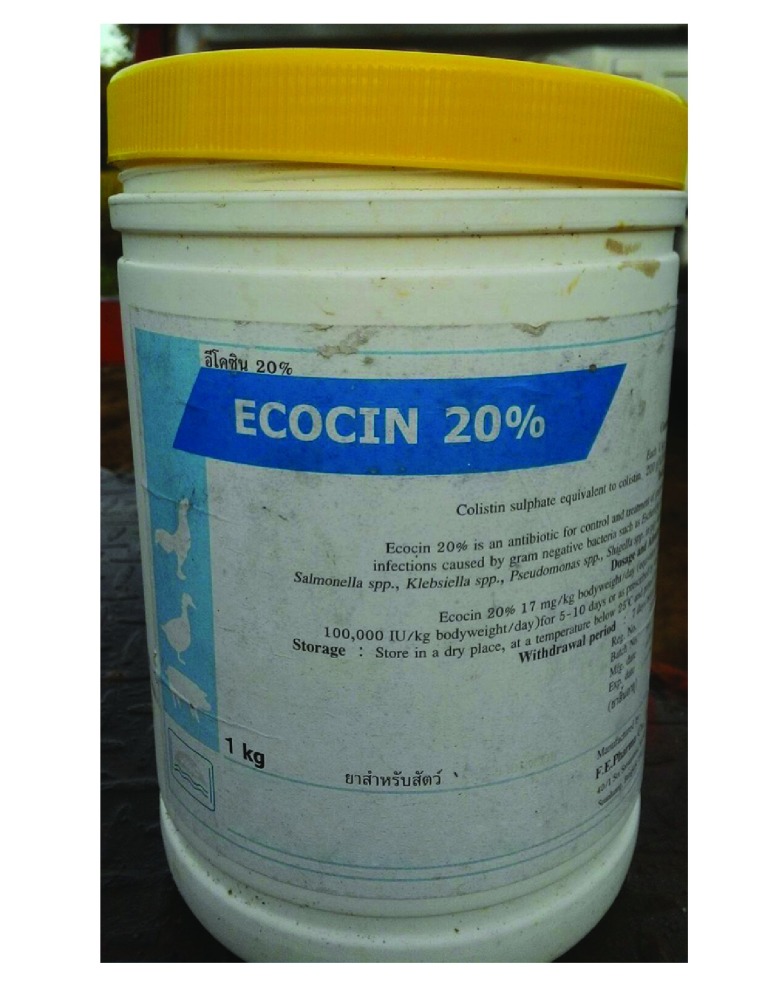
An empty tub, previously used to store colistin powder, on a rural chicken farm, Thailand, 2016

Note: The tub, which measured 14 cm in diameter and 21 cm in height, had previously contained 1 kg of antibiotic powder. Distributor’s details have been masked.

The antibiotic regimen used, for routine prophylaxis, to raise 14 000 of the 3-kg meat chickens for company B was observed (Table 1). In this regimen, which included amoxicillin, colistin, doxycycline, oxytetracycline and tilmicosin, the mean total weight of antibiotics used per chicken was 303 mg. The owners of the three farms using this regimen, which was recommended by company B, stated that they followed the regimen strictly. All antibiotics were mixed with drinking water and distributed via a pipe system (Fig. 1). Empty containers that had contained the antibiotics needed for the regimen, i.e. bottles that had each contained 200 mL of tilmicosin solution, packages that had each contained 500 g of amoxicillin or doxycycline and tubs that had each contained 1000 g of colistin or oxytetracycline, were observed on each of the three farms that reportedly used the regimen (Fig. 2). Although chickens were allocated 41 days on these three farms, antibiotic use was halted on day 31. The 10 days without antibiotics represented an attempt to eliminate antibiotics from the chicken meat reaching consumers.

**Fig. 3:**
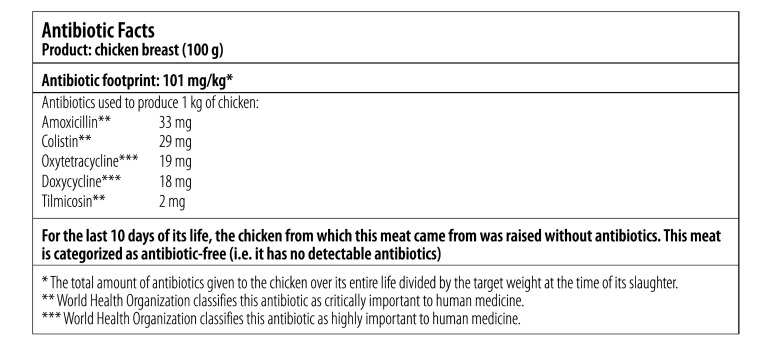
A mock-up of a retail label, for chicken meat, that includes information on the meat’s antibiotic footprint

According to the owners of the farms using routine antibiotic prophylaxis, by the time that a chicken was ready to be transported to company B, it would have been given 101 mg antibiotics – that is, 33 mg of amoxicillin, 29 mg colistin, 19 mg oxytetracycline, 18 mg doxycycline and 2 mg tilmicosin, per kg final weight. In Fig. 3, we illustrate a mock-up of retail packaging for chicken meat, from company B, that includes information about antibiotic use.

Each year, over eight cycles, a farm with a capacity of 14 000 chickens could raise 112 000 chickens for company B and use 34 kg of antibiotics (Table 1). If 62% of the 1.4 billion meat chickens raised in Thailand in 2016 had been raised without the use of any antibiotics and 38% had been given only the antibiotic prophylaxis we observed in the surveyed farms producing for company B, the total amount of antibiotics used on those 1.4 billion chickens would have been about 161 tonnes.

## Discussion

Amoxicillin, colistin, doxycycline, oxytetracycline and tilmicosin were used routinely, for prophylaxis, on three of the farms we surveyed. For human medicine, according to WHO, amoxicillin is a critically important antimicrobial, colistin is one of the highest priority critically important antimicrobials and doxycycline and oxytetacycline are highly important antimicrobials.^14^ Although tilmicosin is not used in humans, it is considered analogous to critically important human medicines in the macrolide group and, therefore, could drive resistance to these drugs.^14^ Although amoxicillin, colistin, doxycycline, oxytetracycline and tilmicocin are all important to human medicine, the United States Food and Drug Administration has approved their use in food-producing animals.^4^^,^^5^^,^^14^ By 2016, however, colistin had never been marketed for use in animals in the United States of America.^15^

Colistin is currently considered to be the last defence against multidrug-resistant bacteria, especially those resistant to carbapenem antibiotics.^5^ The increasing use of colistin in livestock production in China may have resulted in high selective pressures leading to the acquisition of the colistin-resistance *mcr-1* gene by *Escherichia coli*.^16^ China recently banned the use of colistin as a growth promoter and released a mandate controlling the drug’s use in the treatment of disease in animals.^17^ The European Union has set a target for colistin use in food-producing animals, of less than 5 mg per population correction unit,^15^^,^^17^ that might be considered as a primary target for policy-makers in all low- and middle-income countries. If this target were to be made mandatory in Thailand, the chicken farms producing for company B would have to reduce their overall use of colistin by at least 83%, i.e. from a value of at least 28 mg per kg final weight, as recorded in the present study, to one below 5 mg per kg final weight. In Thailand, increases in the incidence of community-acquired antimicrobial-resistant bacterial infection^8^^,^^18^ and colistin-resistant bacteria in the stools of healthy people^19^ have been observed. These observations may be at least partially attributable to the routine exposure of millions of Thai chickens to colistin and several other medically important antibiotics.

There appear to be no published data on antibiotic use in European chickens that could be validly compared with the routine antibiotic use, of about 101 mg per kg final weight, that we recorded, in Thailand, in the farms producing chickens for company B. The antibiotic use we recorded does, however, fall within the wide range of corresponding values previously reported for meat, of all types, produced in 30 European countries, i.e. 2.9–434.2 mg per population correction unit.^5^ The Dutch government has restricted macrolide use in Dutch farms that raise chickens for meat and, between 2014 and 2015, British farmers reduced antibiotic use by 27% when raising chickens for meat.^5^ Better relevant information from high-income countries, e.g. on overall use or sales of antibiotics in livestock production and on the drug classes of the antibiotics used on each species of livestock, would be useful. Such information could be used to see if, as generally suspected, antibiotic use in livestock production is on a larger scale and a greater problem in low- and middle-income countries than in high-income countries.

Thai meat and meat products labelled “antibiotic-free” should contain no – or, at least, no detectable, residual antibiotics. Consumers may believe, often incorrectly, that meat so labelled comes from animals that were raised without any antibiotics. Although the use in livestock production of antibiotics that are important in human medicine may pose a particular challenge to human health, the definitions of an antibiotic that is important in human medicine vary from country to country.^20^ A qualitative approach to meat labelling, e.g. where meat may be labelled “antibiotic-free” or “raised without antibiotics” or have labels with no antibiotic-related information, could lead to false or misleading claims.^20^ We believe it would be better to inform consumers and policy-makers of the full details of the antibiotics used while producing meat. The addition, to labels, of information on the carbon footprint of food products has been found to have an impact on consumers’ judgments.^12^^,^^13^ It remains unclear whether the addition, to labels, of information on the antibiotic footprint of meat products would also have an impact on consumers’ judgment, on the consumers’ willingness to pay and, ultimately, on antibiotic use in the whole agricultural industry. Once global targets for antibiotic use in livestock production are agreed, it may be simpler and easier, for consumers who are deciding which meat to buy, to use retail labels to indicate which products meet or exceed those target values, e.g. via so-called traffic-light food labelling.^5^^,^^21^^–^^23^

Our study has several limitations. By ignoring therapeutic use of antibiotics, we will have underestimated the antibiotic use on our study farms. We were pleased to see no use of antibiotics as growth promoters. However, it is possible that antibiotic use in animal agriculture in low- and middle-income countries has been shifting from growth promotion to routine prophylactic use and therapeutic use. Even if successful, attempts to discourage the use of antibiotics as growth promoters may not have led to an overall decline in antibiotic use in livestock production. In the Netherlands, use of antibiotics as growth promoters fell markedly between 1999 to 2006, but multiple measures were later needed to lower the total antibiotic use in animals, from the peak of about 550 tonnes in 2007 to about 250 tonnes in 2012.^24^ Our extrapolation of results from just eight farms to the whole of Thailand was crude and subject to many sampling biases. We also assumed that the mean weight of a chicken at slaughter was the target weight that had been set for the farmers. Our estimate of the total amount of antibiotic used in raising Thai chickens for meat is unlikely to be accurate.

We propose that the extent and nature of antibiotic use in animals should be officially and openly reported by each farm, sector and country. Also, the labels on animal products should contain information on antibiotic footprints that is similar to that already given, on some products, on carbon footprints.^12^^,^^13^ These measures may encourage a reduction in antibiotic use globally and lead to a reduction of drug-resistant bacteria.
